# Hematopoietic and mesenchymal stem cells: polymeric nanoparticle uptake and lineage differentiation

**DOI:** 10.3762/bjnano.6.38

**Published:** 2015-02-05

**Authors:** Ivonne Brüstle, Thomas Simmet, Gerd Ulrich Nienhaus, Katharina Landfester, Volker Mailänder

**Affiliations:** 1 Max-Planck-Institute for Polymer Research, Ackermannweg 10, 55128 Mainz, Germany; 2 Institute of Pharmacology of Natural Products & Clinical Pharmacology, Ulm University, Helmholtzstraße 20, 89081 Ulm, Germany; 3 Institute of Applied Physics, Karlsruhe Institute of Technology (KIT), Wolfgang-Gaede-Straße 1, 76131 Karlsruhe, Germany; 4 Institute of Toxicology and Genetics (ITG), Karlsruhe Institute of Technology (KIT), Hermann-von-Helmholtz-Platz 1, 76344 Eggenstein-Leopoldshafen, Germany; 5 Department of Physics, University of Illinois at Urbana-Champaign, Urbana, Illinois 61801, USA; 6 3rd Department of Medicine (Hematology, Oncology, and Pneumology), University Medical Center of the Johannes Gutenberg-University Mainz, Langenbeckstraße 1, 55131 Mainz, Germany

**Keywords:** cytokine secretion, differentiation, hematopoietic stem cells, mesenchymal stem cells, nanoparticles

## Abstract

The combination of stem cell therapy and nanoparticles promises to enhance the effect of cellular therapies by using nanocarriers as drug delivery devices to guide the further differentiation or homing of stem cells. The impact of nanoparticles on primary cell types remains much more elusive as most groups study the nanoparticle–cell interaction in malignant cell lines. Here, we report on the influence of polymeric nanoparticles on human hematopoietic stem cells (hHSCs) and mesenchymal stem cells (hMSCs). In this study we systematically investigated the influence of polymeric nanoparticles on the cell functionality and differentiation capacity of hHSCs and hMSCs to obtain a deeper knowledge of the interaction of stem cells and nanoparticles. As model systems of nanoparticles, two sets of either bioinert (polystyrene without carboxylic groups on the surface) or biodegradable (PLLA without magnetite) particles were analyzed. Flow cytometry and microscopy analysis showed high uptake rates and no toxicity for all four tested particles in hMSCs and hHSCs. During the differentiation process, the payload of particles per cell decreased. The PLLA–Fe particle showed a significant increase in the IL-8 release in hMSCs but not in hHSCs. We assume that this is due to an increase of free intracellular iron ions but obviously also depends on the cell type. For hHSCs and hMSCs, lineage differentiation into erythrocytes, granulocytes, and megakaryocytes or adipocytes, osteocytes and chondrocytes, was not influenced by the particles when analyzed with lineage specific cluster of differentiation markers. On the other hand qPCR analysis showed significant changes in the expression of some (but not all) investigated lineage markers for both primary cell types.

## Introduction

Interaction of different stem cell types with nanomaterials has been of interest lately for several reasons. One of the reasons is that nanoparticles (materials with dimensions well below the micrometer range) have been proposed for labeling of primary cells, including stem cells, in order to study homing and engraftment [1–2] or to deliver drugs. Labeling with iron-containing particles provides the possibility to track the cell fate in vivo by using noninvasive magnetic resonance imaging (MRI). Superparamagentic iron oxide particles (SPIONs) are used for this purpose but also gadolinium-loaded nanotubes can be rendered magnetic with the objective of keeping the stem cells at a desired place in the human body [3]. More advanced approaches address the delivery of drugs or other agents into stem cells [4–5], as stem cells are regularly processed ex vivo and are therefore amenable to further treatment. Here, nanomaterials could provide a means of manipulating the fate of the stem cells, for example, by influencing migration in vivo by (over-)expression of homing receptors or influencing stem cell differentiation by providing the cells with an intracellular depot of a drug or a nucleic acid construct with slow release kinetics. Thus, the intended nanoparticles should be tested for toxicity and the nanomaterial as a carrier ideally should not influence cellular functions itself, that is, only the payload should exert such an effect. Once introduced into the system, toxicity can occur from the nanoparticles themselves or from the associated components of the nanoparticles that might be released during degradation in vivo. In addition to potentially causing toxicity after cellular uptake, nanoparticles could also alter cellular functions such as the differentiation potential or secretion profile of, for example, cytokines. The evaluation of these risks is a milestone for the combination of nanomaterials with stem cells. One of the most widely studied stem cell populations is undoubtedly the human hematopoietic stem cell (hHSC), which has been successfully used for many years for the treatment of leukemia and lymphoma as well as for other, non-malignant diseases such as aplastic anemia. Toxicity and cell functionality studies with hHSCs and nanoparticles are rare and have been performed only for a few different particle/material combinations [6–9]. As these were not studied side-by-side, comparison of the results is not commensurable.

Human mesenchymal stem cells (hMSCs) are a promising tool for cell-based therapeutic strategies because they can be isolated and expanded to high numbers in vitro, they possess the ability to self-renew and the differentiation capacity towards lineages are interesting for regenerative therapy (reviewed in [10]). Furthermore, they are hypoimmunogenic, which makes them suitable for allogeneic transplantations and they can even have immunosuppressive functions [11–12]. hMSCs are fibroblast-like cells that were first described by Friedenstein and colleagues [13] and can be obtained from various tissues including bone marrow [14], adipose tissue [15] and most connective tissues [16]. Due to their ability to differentiate towards adipocytes, chondrocytes and osteocytes [14], these cells are also of a great interest for tissue engineering approaches (e.g., for defects of bone or cartilage). Over 100 clinical trials employing hMSCs for regenerative medicine, for instance, after stroke and myocardial infarction [17], demonstrate that the clinical use of these cells is of utmost interest.

Therefore, the combination of nanoparticles with these two stem cell types derived from the bone marrow is very promising not only for labelling to monitor biodistribution and migration of stem cells but also to establish the “pharmacokinetics” of such cellular therapeutics. Furthermore, such nanoparticles can be potentially used as carriers for drugs to influence the stem cell fate once they have been delivered into a patient. For all of these applications, the nanoparticle should act only as a delivery platform, and should not negatively influence the stem cell behavior.

## Results

### Particles

Two sets of nanoparticles were chosen for this study: non-functionalized polystyrene (PS) and carboxy-functionalized polystyrene (PS–COOH). PS–COOH particles are biocompatible, but nondegradable particles whereas poly(L-lactide) particles without (PLLA) and with magnetite (PLLA–Fe) are biocompatible and biodegradable. All particles were fluorescently labeled with the fluorescent dye *N*-(2,6-diisopropylphenyl)perylene-3,4-dicarboximide (PMI). The magnetite was encapsulated for magnetic resonance purposes. The polymeric nanoparticles used in this work were obtained by miniemulsion polymerization (non-functionalized and functionalized polystyrene nanoparticles as described in [18]) or by a combination of miniemulsion and emulsion/solvent evaporation techniques (PLLA nanoparticles without and with magnetite, as described in [19–20]). In all cases, SDS was used as a surfactant for the synthesis or formation of the nanoparticles. The nanoparticles were purified from the surfactant excess by dialysis using Amicon Ultra membrane filters with MWCO 100 kDa (Millipore). The main characteristics of the nanoparticles are described in Table 1.

**Table 1:** Characterization of the nanoparticles.

Sample name	Polymer	Particle loading^a^	Functional groups/particle	Diameter [nm]	Zeta potential [mV]

PS–COOH	poly(styrene-*co*- arylic acid)	PMI	8.7 × 10^4^	121	−46
PS	polystyrene	PMI		116	−45
PLLA–Fe	PLLA (140,000 g/mol)	PMI/ magnetite 2.53 mg Fe/mL dispersion		124	−30
PLLA	PLLA (140,000 g/mol)	PMI		131	−29

^a^PMI (fluorescent dye), *N*-(2,6-diisopropylphenyl)perylene-3,4-dicarboximide.

### Uptake and toxicity of polymeric nanoparticles

Before the nanoparticles were used for differentiation studies, cellular uptake and cytotoxicity of the particles were quantitatively determined by flow cytometry.

**Figure 1:**
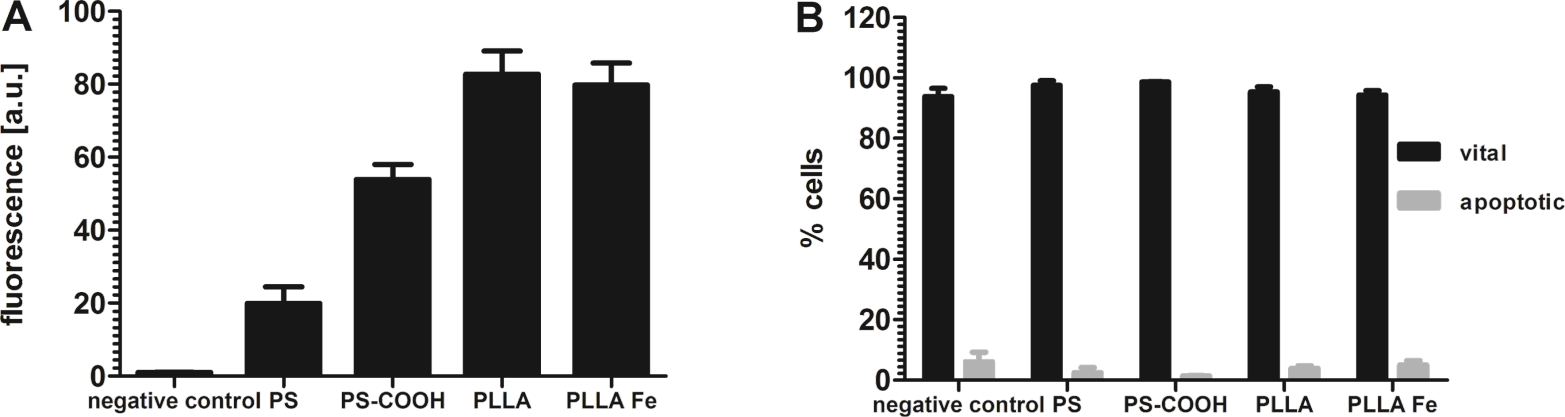
(A) Nanoparticle uptake after 24 h incubation of hMSCs with 300 µg/mL nanoparticles analyzed by flow cytometry. Fluorescence [a.u.] = median fluorescence intensity, arbitrary units. All cells with nanoparticles show a significantly higher fluorescence intensity compared to the negative control (three independent experiments, p < 0.05). (B) Cytotoxicity study of the nanoparticles after 24 h incubation with 300 µg/mL particles analyzed by 7-AAD staining and flow cytometry. Dead cells were not included in the analysis because they constituted less than 1%. The amount of apoptotic cells is given in Supporting Information File 1, Figure S1.

After incubation with 300 µg/mL nanoparticles for 24 h, hMSCs showed a reasonable uptake of polystyrene nanoparticles (PS, Figure 1A). Since here only one population was detected in flow cytometry, the data are given as median fluorescence intensity (MFI).

**Figure 2:**
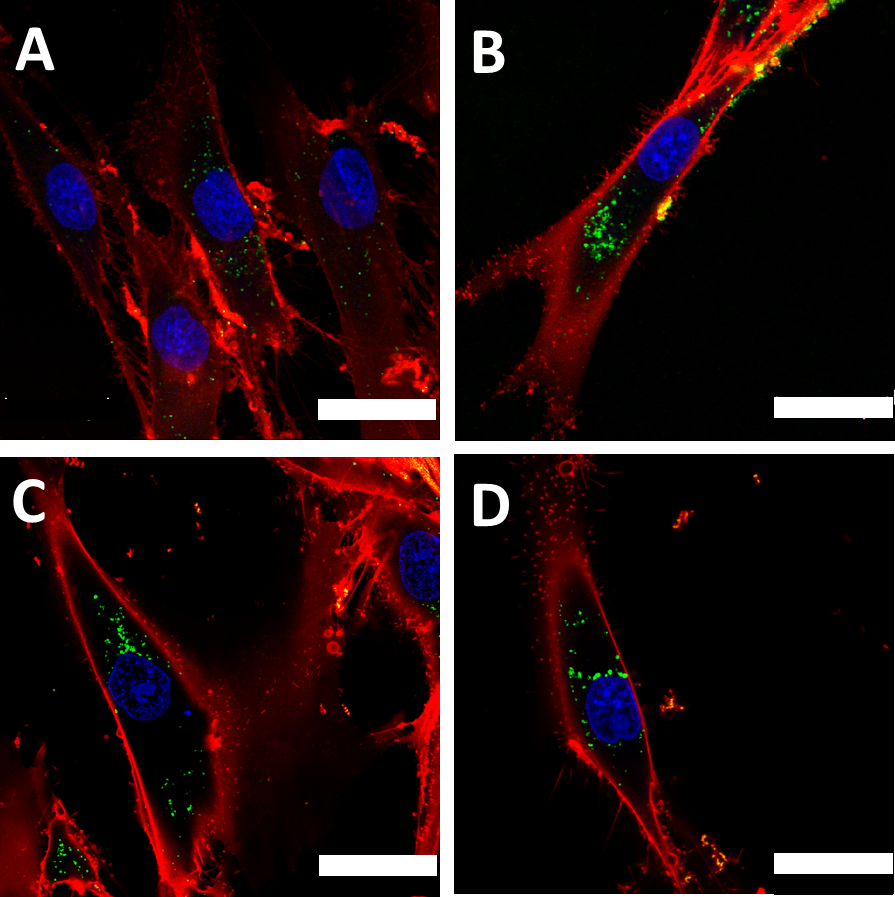
Particle uptake into hMSCs detected by cLSM after 24 h incubation with 300 µg/mL nanoparticles. (A) PS, (B) PS–COOH, (C) PLLA, (D) PLLA–Fe. The cell membrane is stained with CellMask Orange (red), nanoparticles are depicted in green, the cell nucleus is stained with DraQ5 and is pseudo-colored in blue. The white scale bar represents 25 µm.

Functionalization of the nanoparticles with carboxy groups on the surface leads to a 3-fold increase in particle uptake for PS–COOH. The positive effect of surface functionalization on the uptake for hMSCs with carboxy groups has been previously demonstrated [18]. In addition to the good uptake properties, the polystyrene particles did not have any cytotoxic effect (Figure 1B). The biodegradable PLLA nanoparticles also showed good uptake and no cytotoxic effects (Figure 1A,B). This was also true for nanoparticles with iron oxide (magnetite) embedded (PLLA–Fe). Confocal laser scanning microscopy (cLSM) was performed to determine the intracellular localization of the nanoparticles. The images in Figure 2 clearly show that all particle types were indeed internalized by the cells and not only attached to the cell surface.

**Figure 3:**
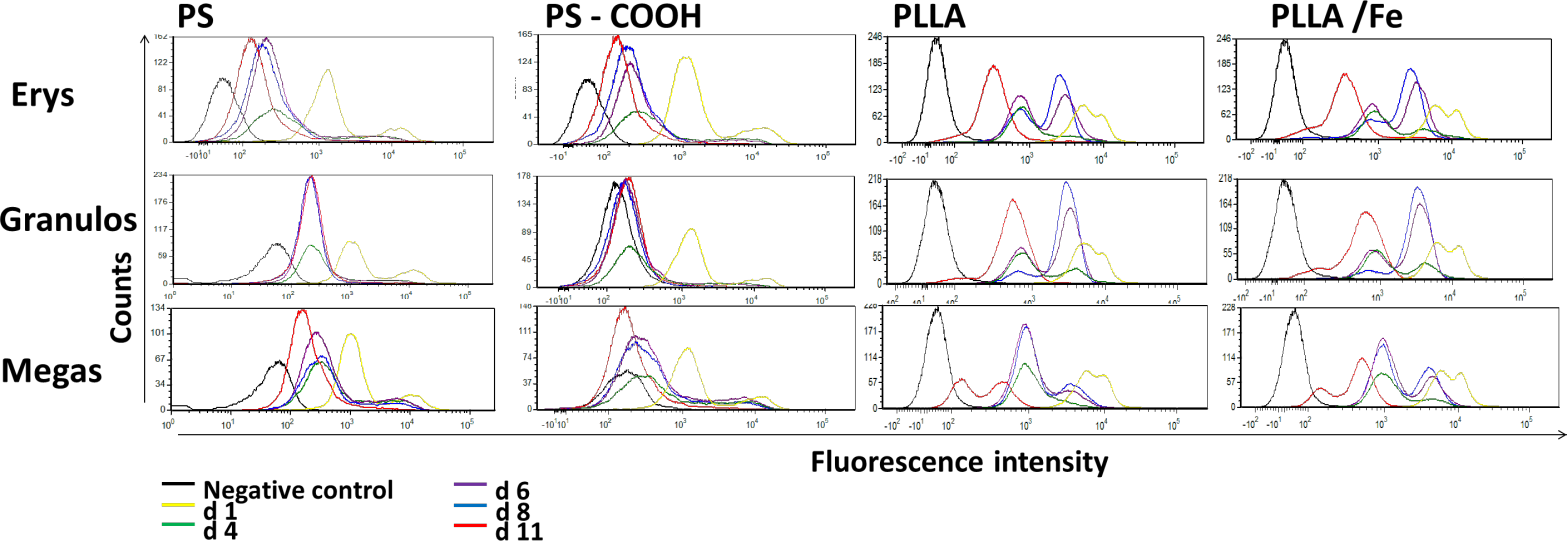
Particle uptake of hHSCs after 24 h incubation with 300 µg/mL nanoparticles. The nanoparticle content was measured every 2 days during differentiation time. Gating was based on FSC/SSC in order to exclude debris. Further gating was based on the differentiation markers for the erythrocyte lineage (CD71, CD235a), granulocytic lineage (CD11b, CD15) and megakaryocytic lineage (CD41a, CD42b).

hHSCs showed a reasonable uptake of the polystyrene nanoparticles after incubation with 300 µg/mL nanoparticles for 24 h and an even greater uptake for the PLLA particles. All four particles showed a double population in the fluorescence histogram for hHSCs, pointing to the possibility that there are still several subsets within the CD34+ selected hHSCs that differ in their ability to take up nanoparticles (Figure 3).

Notably, although two populations of hHSCs were detected, all cells in the two populations showed a fluorescence intensity above the background level of the negative control, demonstrating that cells in both populations had taken up nanoparticles. The cLSM results for the hHSCs clearly showed that particles were incorporated (Supporting Information File 1, Figure S2).

During the differentiation time of 11 days, the cells in all lineages showed a pronounced loss of the fluorescence signal and therefore of nanoparticles. This was very much expected as the proliferation of hHSCs during the growth factor induced differentiation will decrease the amount of nanoparticles per cell division by a factor of 1/2. For the PLLA particles, the remaining amount was significantly higher than the negative control even after 11 days. In the case of the PS particles, the remaining number of nanoparticles after 11 days was lower, and the PS–COOH were indistinguishable from the negative control for megakaryocytic and also from the granulocytic differentiation. The PS and as well the PLLA particles did not show any detectable toxicity (Supporting Information File 1, Figure S3).

### Cytokine secretion of hMSCs and hHSCs

**Figure 4:**
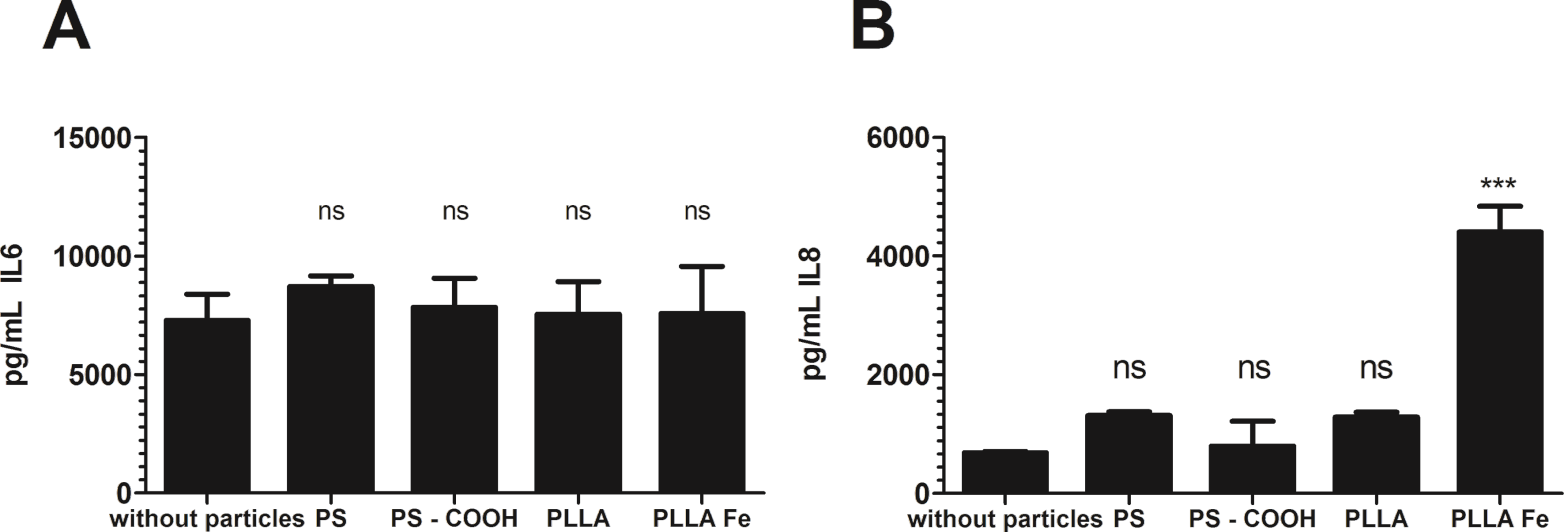
Cytokine secretion of hMSCs treated with different nanoparticles: (A) IL-6, (B) IL-8. hMSCs were incubated with 300 µg/mL particles for 24 h, cytokine detection was performed after 5 days of cultivation with a HTRF assay by analyzing the cell culture supernatant. p > 0.05 ns, p < 0.001 ***.

To determine if the polymeric nanoparticles have an impact on the cell functionality, IL-6 und IL-8 were chosen as they have been reported to be secreted by hMSCs [21–22]. The concentration of these two cytokines was measured with a HTRF^®^ assay. Cells were incubated with 300 µg/mL nanoparticles for 24 h, and excess particles were washed away. Then the cells were incubated in fresh medium for 5 days and the supernatant was used for the HTRF^®^ measurements. The polystyrene particles did not influence the IL-6 or IL-8 secretion (Figure 3). The lone PLLA particles also showed no influence on the cytokine secretion. In contrast, the PLLA–Fe particles dramatically increased the IL-8 release while the IL-6 secretion was not altered (Figure 4).

For hHSCs, cell functionality was tested by quantitative analysis of the IL-8 secretion. Cells were incubated with 300 µg/mL nanoparticles for 24 h and afterwards cells were washed three times with PBS^−^ and cultivated for 5 days in a medium with SCF and Flt. IL-8 secretion was measured in the supernatant at the end of this cultivation period. The secretion level of IL-8 was not significantly altered in the presence of the polymeric nanoparticles (Supporting Information File 1, Figure S4).

### Differentiation behavior under nanoparticle influence

#### Cytochemical staining of hMSCs

**Figure 5:**
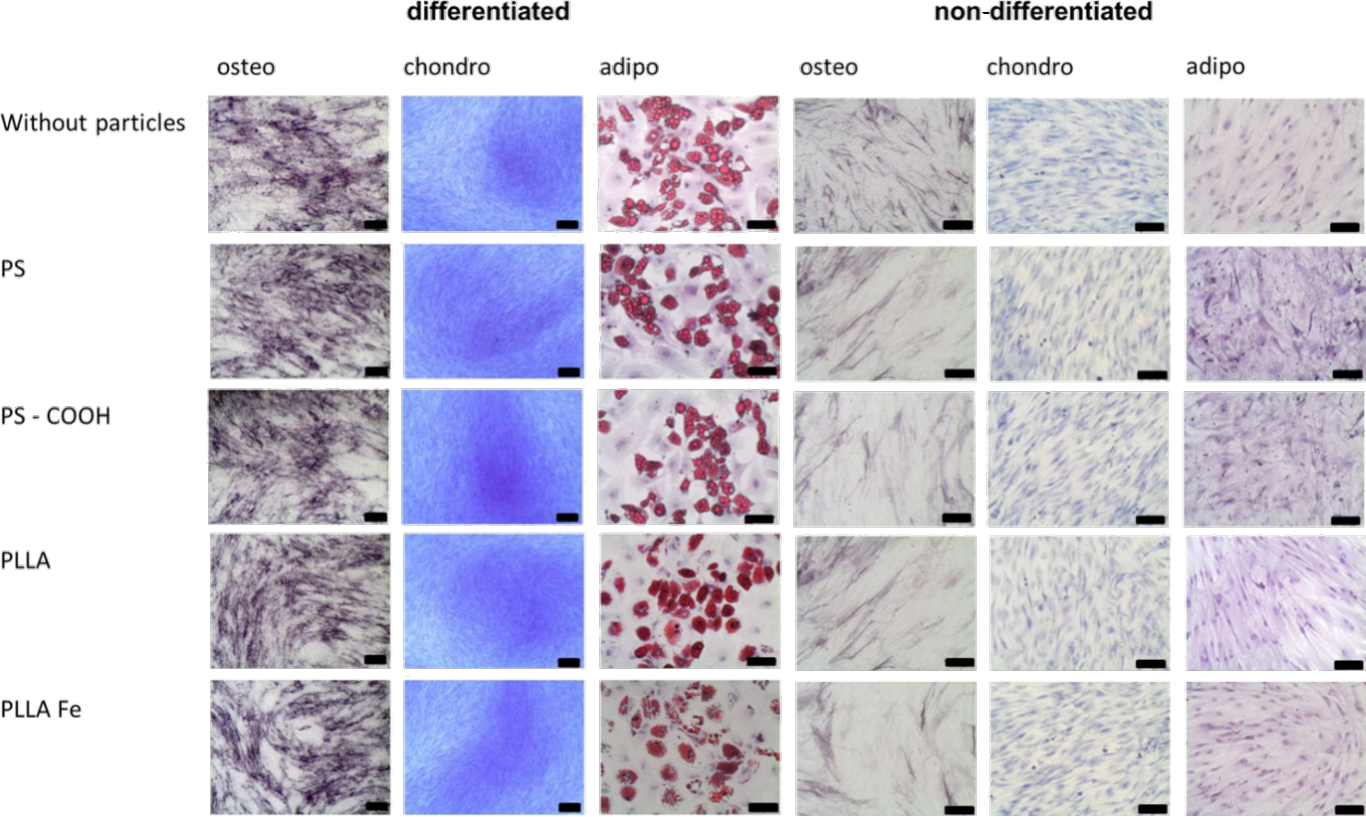
Cytochemical staining to determine the differentiation of hMSCs incubated with different nanoparticles (300 µg/mL nanoparticles, incubation time 24 h, before inducing differentiation). Osteogenic differentiation was demonstrated by alkaline phosphatase activity, chondrogenic differentiation by methylene blue staining of the extracellular matrix, and adipogenic differentiation by Oil-Red O staining of the lipid droplets. Samples without particle incubation (without particles) served as controls. The black scale bar represents 200 µm (except for adipo and non-differentiated samples, 100 µm).

For analyzing the particle influence on the differentiation capacity of hMSCs, the cells were incubated with 300 µg/mL nanoparticles for 24 h before starting the differentiation by providing the adequate differentiation medium. After 24 days, osteogenic and chondrogenic differentiation was analyzed by detecting alkaline phosphatase activity or methylene blue staining, respectively. Detection of adipogenic differentiation was performed after 4 weeks with Oil-Red O staining. In the presence of the nanoparticles, the differentiation into the three investigated lineages was not affected, as determined by cytochemical staining (Figure 5).

The detection of the extracellular matrix for the chondrogenic differentiation, the alkaline phosphatase activity for the osteogenic differentiation, as well as the lipid droplets for the adipogenic differentiation showed the same characteristics as the sample without nanoparticles. To investigate if the presence of polymeric nanoparticles can induce differentiation without the use of specific media, the same differentiation experiment was performed for differentiation media, but the cells were kept in α-MEM for the entire time. These samples were labeled as “non-differentiated”. The particles also show no indication of the onset of the differentiation process in the absence of the specific differentiation media. No specific differentiation evidence could be detected with the cytochemical staining.

#### qPCR analysis of the expression of the different lineage markers for hMSCs

**Figure 6:**
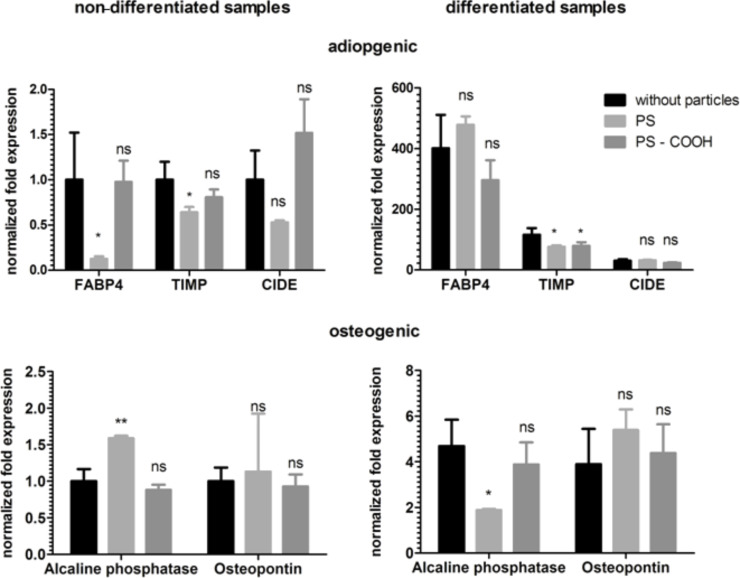
Influence of polystyrene nanoparticles (PS and PS–COOH) on the expression of adipogenic and osteogenic marker genes in hMSCs. Expression was analyzed with qPCR, using GAPDH and B2M as an internal control. The normalized fold expression was calculated with the ΔΔC_T_ method, assigning the non-differentiated sample without particle as a control. p > 0.05 ns, p < 0.05 *, p > 0.01 **; FABP4: fatty acid binding protein 4; TIMP: tissue inhibitor of metalloproteinase; CIDE: cell death inducing DFFA-like effector.

To assess whether the polymeric nanoparticles had an influence on the expression of marker genes during the differentiation, qPCR analysis on cDNA from RNA extracted from differentiated and non-differentiated samples was performed. The non-functionalized PS particle significantly decreased the expression of the adipogenic markers FABP4 and TIMP in the non-differentiated samples. The carboxy functionalized PS–COOH nanoparticles, in contrast, had no influence on the expression of the marker genes in the non-differentiated samples. For the differentiated samples, both particles showed a significant decrease of the expression level of TIMP, and the other two lineage markers were not affected (Figure 6).

**Figure 7:**
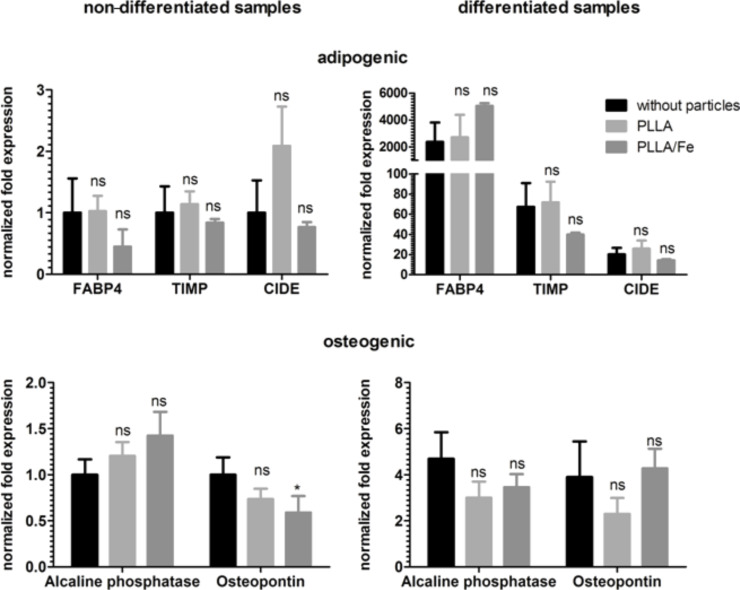
Influence of polylactide nanoparticles (PLLA and PLLA–Fe) on the expression of adipogenic and osteogenic marker genes in hMSCs. Expression was analyzed with qPCR, using GAPDH and B2M as internal controls. The normalized fold expression was calculated with the ΔΔC_T_ method, assigning the non-differentiated sample without nanoparticles as a control. p > 0.05 ns, p < 0.05 *.

The presence of PLLA and PLLA–Fe particles during the adipogenic differentiation showed no impact on the expression level of these lineage markers (Figure 7).

The expression of the osteogenic lineage marker ALPI II is influenced by the presence of the PS nanoparticles but not by the PS–COOH nanoparticles (Figure 6). Interestingly, the expression of ALPI II is significantly upregulated in the non-differentiated samples, and in the differentiated samples, there is a significant decrease in ALPI II expression. The expression of osteopontin was not altered in the presence of polystyrene nanoparticles. In the case of the PLLA particles, only the expression of osteopontin is significantly reduced in the non-differentiated samples for the PLLA–Fe particles. Although statistically different, the relevance of an expression level of approximately 0.6 compared to the control is not impressive and may not be strong enough to divert further development. All other expression values are not affected by the presence of these particles (Figure 7).

#### Differentiation in flow cytometry for hHSCs

Differentiation of hHSCs in three lineages was determined after incubation with 300 µg/mL nanoparticles with CD marker expression analysis and qPCR. All three lineages showed no alteration in CD marker expression after the incubation with all four polymeric nanoparticles (Supporting Information File 1, Figures S5,S6). All CD markers were clearly expressed at a higher level after the differentiation, which indicated a correct lineage differentiation.

#### qPCR analysis of the expression of the different lineage markers for hHSCs

**Figure 8:**
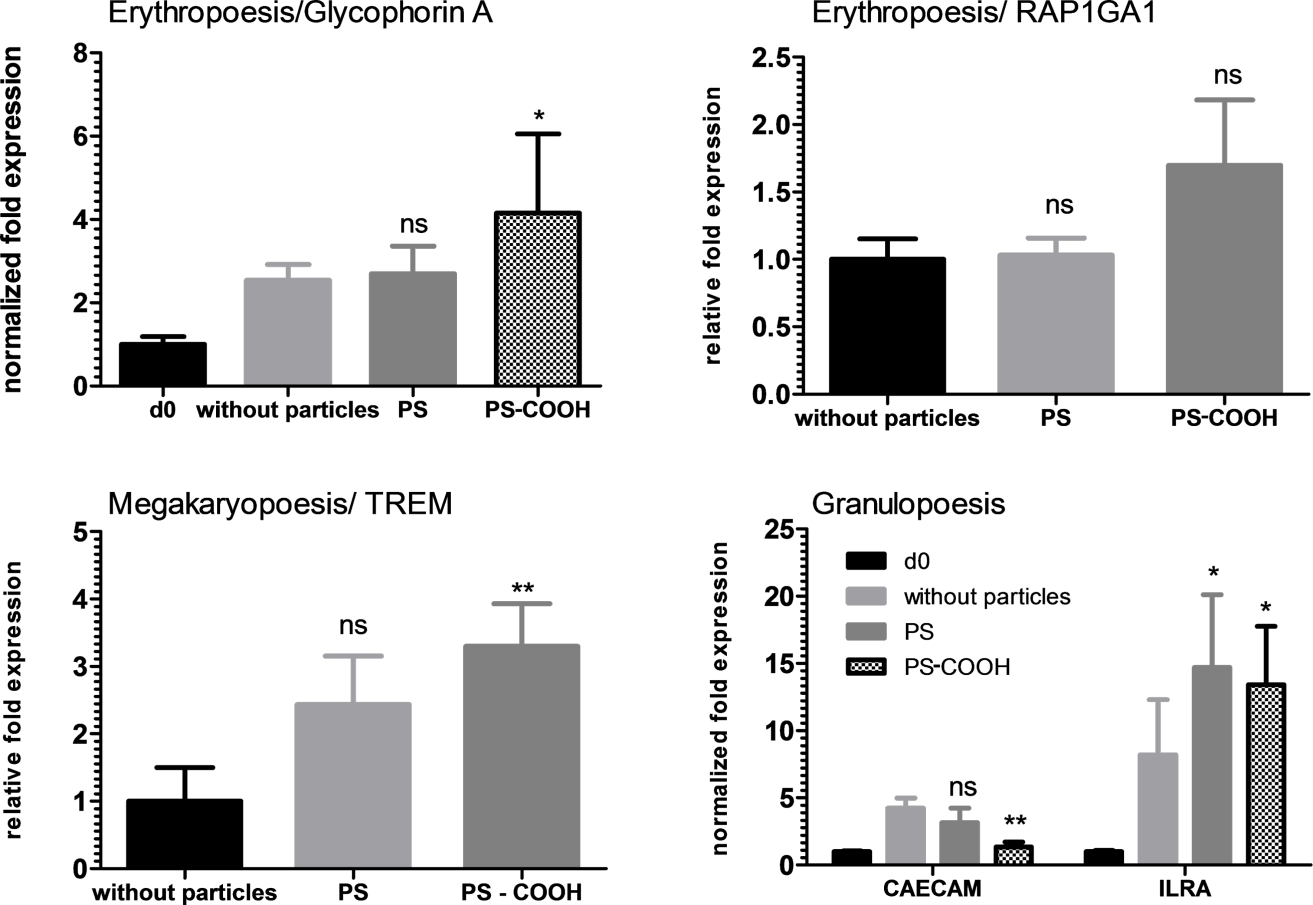
qPCR results of polystyrene particles in hHSCs. Carboxy-functionalized polystyrene particles PS–COOH showed a slight increase in glycophorin A, TREM and ILRA transcription, while a decrease for CAECAM was observed. For pure polystyrene, only ILRA was increased.

When analyzing at the RNA level for differentiation markers for hHSCs, several changes in the expression level were observed. In all three differentiation lineages, the polystyrene particles significantly altered the expression of some but not all differentiation markers. The PS particles only caused a significant increase in the expression of the granulopoetic marker ILRA (Figure 8). Although we did not carry out further analysis at the protein level, it seems to be worthwhile to perform an ELISA for ILRA where nanoparticles are used to label hHSCs or granulocytes or where nanoparticles are used to deliver drugs or other molecules into hHSCs or granulocytes.

**Figure 9:**
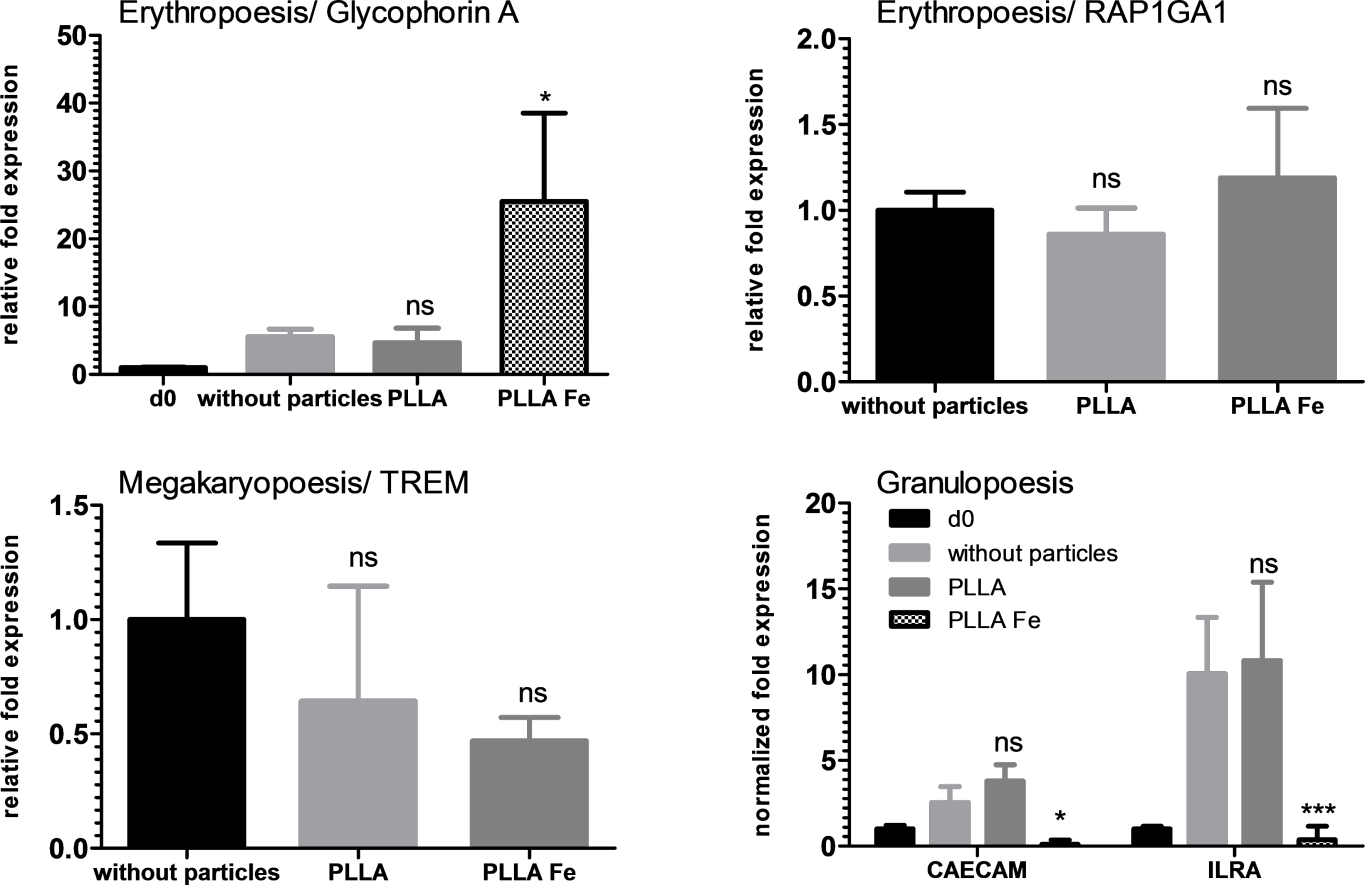
qPCR results of the polylactide particles in hHSCs. For glycophorin A, a significant increase could be detected when PLLA–Fe particles were used. On the other hand, the granulocytic markers CAECAM and ILRA were suppressed with PLLA–Fe particles.

The PS–COOH particles altered the expression of marker genes of all three lineages. The pure PLLA particles did not influence the expression of any of the investigated marker genes (Figure 9). In contrast, the PLLA–Fe particles had a strong influence on some marker genes. The expression of glycophorin A was significantly increased by the presence of the PLLA–Fe particles, whereas the expression of both granulopoetic markers was significantly suppressed.

## Discussion

Although nanoparticles are proposed as useful drug and DNA or siRNA delivery vehicles (which could potentially change the fate of stem cells), they could also influence the cellular fate of stem cells with a potentially devastating effect. While hMSCs have been used in many studies, only a limited number of studies with these cells have been undertaken [1]. Cells labeled with superparamagnetic nanoparticles can be tracked after transplantation using MRI methods [23]. This approach allows for a deeper knowledge about the biological distribution of the engrafted cells in the body to be gained. While some nanoparticles may have no or little effect on viability and differentiation [24], some nanoparticles may tend to guide the differentiation towards a desired lineage without the need of specific growth factors [25]. As a second step, the cells could be loaded with drugs containing particles for enhancing the differentiation in one direction or improving the graft efficiency [26–27]. Before using these complex nanoparticle systems, all potential risks (e.g., toxicity and alteration in cell functionality) concerning the material, surface functionality, etc. should be investigated. Additionally, even for industrial applications (e.g., coatings in automotive and other types of industries) toxicology studies are warranted. In this study, we demonstrated that polystyrene and polylactide particles are taken up not only by hMSCs, but also at a reasonable amount by hHSCs. This is itself interesting as hHSCs have no phagocytotic properties, and the endocytotic processes in hHSCs are not known for a high turnover rate. Flow cytometry analysis showed that the particle uptake differs between the two materials, polystyrene and polylactide. Polylactide particles are taken up at a higher concentration in both cell types compared to polystyrene particles, demonstrating the comparative investigation of different materials. The surface functionalization of the PS–COOH particles led to an increase of the particles in hMCS, but this was less effective in hHSCs. The hHSCs showed a great loss in particle payload during all three lineage differentiations. This is likely due to the high proliferation rates during the differentiation process. In other studies, this dilution due to proliferation activity of the cells has clearly been shown [28–29]. In the literature, the interaction of hHSCs and nanoparticles is poorly investigated, probably due to the lack of adequate material in many laboratories. There are some studies with particles containing iron for cell labeling for MRI measurements. In different studies with the MRI contrast agent Ferridex^®^ (iron oxide with a dextran shell), no toxicity and normal differentiation behavior was shown for hHSCs [6–8]. A study with diverse inorganic nanoparticles of different sizes showed distinct toxicity for some particles (cobalt, antimony oxide) as well as some negative influence on the differentiation process [9]. For polymeric nanoparticles and hHCSs, there are no studies currently available to the authors’ knowledge. Since hMSCs constantly secreted IL-6 and IL-8 [21–22], we therefore investigated these two cytokines. Whereas the secretion of IL-6 was not influenced at all by the polymeric nanoparticles, the secretion of IL-8 was dramatically increased in the presence of the PLLA–Fe particles in hMSCs. This is likely because of the release of iron ions from the particles. Free iron ions within the cell can lead to an increase in oxidative stress [30–32], and a higher level of reactive oxygen species (ROS) can lead to an increased release of IL-8. The observation of an increased IL-8 release has also been reported for silver ions/nanoparticles [33] with hMSCs. For hHSCs, no increase of IL-8 release was observed.

Clearly, the effect on differentiation of hMSCs should be investigated separately from toxicity as Liu et al. demonstrated that even for toxic nanoparticles, differentiation may not be affected [34] while Hou et al. showed that TiO_2_ nanoparticles do affect viability and differentiation [35]. To explore whether the particles could disrupt the differentiation process, the differentiation efficiency was qualitatively and quantitatively analyzed. In the case of hMSCs lineage differentiation, this study clearly showed that in the presence of all four particles, adipogenic, chondrogenic and osteogenic differentiation characteristics were correctly developed when cytochemical staining was performed. In the case of adipogenic differentiation, lipid droplets could be visualized with Oil-red O, the extracellular matrix developing during chondrogensis could be detected with methylene blue staining and, in the case of osteogenic differentiation, the activity of alkaline phosphatase was clearly visible. For an application of nanoparticles in cell-based therapies it is also important that the hMSCs do not lose their self-renewal potential without undergoing differentiation. In this work, we did not see any particle-induced differentiation by itself.

The qualitative analysis of hHSC differentiation showed that the expression of lineage specific CD markers for hHSCs was not altered in the presence of the particles. However, when the expression levels of several differentiation markers was analyzed by qPCR, the expression of some but not of all markers were affected. Especially interesting is the high expression of glycophorin A, which indicates that the erythrocyte differentiation is enhanced with PLLA–Fe particles. This can be explained by the fact that iron in this case is an essential compound for erythrocyte differentiation and, therefore, enhances proliferation in this direction, while other lineages do not require it and it may even have a negative effect such as producing reactive oxygen species (ROS).

For hMSCs, despite of detecting no obvious difference between treated and untreated samples with cytochemical staining, the pPCR analysis showed some alteration in the expression level of the genes investigated. The influence on the expression of different marker genes varied between the particles. Whereas both polystyrene particles led to some down regulation of the adipogenic markers FABP4 and TIMP, all of the polylactide particles did not have an influence on these adipogenic markers. The adipogenic marker CIDE 3 was not influenced by all of the particles. With regards to the osteogenic markers, the situation is similar: the non–functionalized PS particle affects the expression of ALP in the non–differentiated samples as well as in the differentiated, whereas the carboxy-functionalized PS–COOH and PLLA did not influence the expression of all osteogenic markers. The PLLA–Fe particle has some impact on the expression of another osteogenic marker, osteopontin. Taken together this implies that a strong influence on the differentiation of hMSCs is unlikely for the nanoparticles investigated.

Therefore, for hMSCs as well as for hHSCs, longer in vivo studies are crucial to determine if the influence seen in the comparably short in vitro assays also has a functional consequence (e.g., for engraftment after transplantation or cell dose needed for a stable engraftment).

In the complex process of hMSC and hHSC differentiation, it is hard to determine at which point the particle influence takes place. As we observed some gene expression changes, a closer look at the molecular level of the interaction between nanoparticles and cells should be the aim of future research to obtain more information about the use of nanoparticles. Furthermore, a closer look into the signaling pathways and how they are affected by nanoparticles is a challenging but necessary task for further understanding of the underlying processes.

## Conclusion

The present study clearly showed that hMSCs and hHSCs are able to take up different polymeric particles at a reasonable concentration without showing any signs of toxicity. The surface functionalization of the polystyrene particle with carboxy groups did not improve the cellular uptake for hHSCs, while for hMSCs, this effect was confirmed. The secretion of IL-8 as a demonstration of cell functionality was not altered in the presence of either polystyrene or polylactide particles for hHSCs, but was for hMSCs. During the differentiation process, the cells showed a significant reduction in nanoparticle concentration due to high proliferation rates. The differentiation into the respective investigated lineages showed no alteration when analyzed by qualitative methods. However, the qPCR analysis of some lineage markers showed alterations in the expression level of some but not all markers for hHSCs and for hMSCs, although the significance of this finding should be further investigated.

## Experimental

### Particle synthesis

Polystyrene particles were obtained via the miniemulsion process as previously described in [18]. Polylactide particles were obtained by a combination of miniemulsion and emulsion/solvent evaporation techniques as previously described in [19–20]. Particle characteristics are given in Table 1.

#### Isolation of human hematopoietic stem cells (hHSCs)

hHSCs were obtained via apheresis from peripheral blood (peripheral blood stem cells, PBSC) for validation purposes of another study after obtaining informed consent. The apheresis product was selected for CD34+ stem cells by the CliniMACS procedure as part of an established routine process in the Stem Cell Laboratory (“Herstellungslabor für Zelltherapie”) of the University Medical Center of the Johannes Gutenberg University, Mainz. The portion of the CD34+ selected PBSC that was not used for validation in the clinical study was approved by the ethics committee of Rhineland-Palatinate to be used for our study (approval number: 837.425.10 (7434)). A second informed consent was obtained from the donors so that the material could be used for our assays. The cells were stored in the gas phase of liquid nitrogen always kept below −140 °C and used fresh for every experiment.

For all experiments, the cells were seeded at a density of 0.5–2 × 10^6^ cells/well in 12-well plates (Greiner Bio) of 3 mL volume. As a basal medium, Iscove's Modified Dulbecco's Medium (IMDM, Invitrogen) supplemented with 10% fetal calf serum (FCS, Invitrogen), 100 units penicillin and 100 µg/mL medium streptavidin (Invitrogen) were used. For cultivation and differentiation, the cells were kept in a humidified incubator with 5% CO_2_ at 37 °C. All experiments were performed with cells incubated with 300 µg/mL nanoparticles for 24 h.

#### Isolation and cultivation of human mesenchymal stem cells (hMSCs)

MSCs were generated from bone marrow aspirations or explanted hips after obtaining informed consent. The study was approved by the ethics committee of the University Ulm, Germany (approval number: 24/11). Primary human hMSCs were generated as previously described [36].

MSC were cultivated in α–MEM (Lonza, Cologne, Germany) supplemented with 20% fetal calf serum (FCS, Invitrogen, Karlsruhe, Germany), 100 units penicillin and 100 µg/mL medium streptavidin (Invitrogen), 1 mM pyruvate (Invitrogen) and 0.6% ciprofloxacin (Fluka, Buchs, Switzerland) in a humidified incubator with 5% CO_2_ at 37 °C.

For particle uptake and cell viability experiments, cells were seeded at a density of 10,000 cells/cm^2^ in 6-well plates (Greiner Bio, Frickenhausen, Germany). After adherence, the cells were incubated with 300 µg/mL nanoparticles for 24 h and analyzed by flow cytometry.

#### Flow cytometry

Particle uptake, cell viability, and CD marker staining were measured with a flow cytometer (FACS Canto II, BD, Heidelberg, Germany). The cells were washed with phosphate buffered saline without calcium (PBS^−^, Invitrogen) and incubated with 28.6 mg/mL 7-aminoactinomycin (7-AAD, Sigma, Munich, Germany) for 15 min in the dark at room temperature or with the respective antibody at 4 °C. The cells were washed again with PBS^−^ and flow cytometry was performed. All flow cytometric analyses were performed with FCS Express (DeNovo Software, Los Angeles, CA, USA). The nanoparticle dye perylenemonoimide (see [18] for structure and other details) was excited with a 488 nm laser, and the signals was acquired using a 530 ± 30 nm filter. 7-AAD or antibody-coupled APC was excited with the 633 nm laser and the signals were acquired using a 660±20 nm filter (7-AAD or APC). The cells were analyzed by gating on a dotplot of the forward/sideward scatter and thereby excluding free particles and cell debris. The nanoparticle uptake was analyzed in a PMI channel histogram, and the 7-AAD signal in a 7-AAD/PMI channel dotplot by setting three gates (vital, apoptotic, dead) and taking the percentage of gated cells in the respective gate. Signals from APC-coupled antibodies were analyzed by overlays of several signals in die APC channel.

#### Confocal laser scanning microscopy (cLSM)

Confocal laser scanning microscopy was performed to validate the intracellular localization of nanoparticles. Images were taken with a Leica SP5 II with a 60× oil immersion objective. The particle dye PMI was excited with the 488 nm laser, and the emission was collected at 510–540 nm. The cell membrane was stained with Cell Mask Orange according to the recommendations of the manufacturer (Invitrogen), and excited with 543 nm laser light and the emission was collected at 565–585 nm. To avoid crosstalk between the channels, emission signals were collected independently in serial mode.

#### Induction of differentiation for hHSCs

In vitro differentiation of hHSCs was performed as previously described [37]. 1.5 × 10^6^ fresh thawed cells were incubated with 300 µg/mL nanoparticles in 3 mL of basal medium for 24 h in 12-well plates. Before inducing differentiation, the cells were washed twice with PBS^−^. The cells were differentiated in 12-well plates in a volume of 3 mL of basal medium, supplemented with lineage-specific cytokines and growth factors (see Supporting Information File 1, Table S1, all from R&D, USA). After 11 days, 1 × 10^6^ cells were used for RNA extraction, and 0.5 × 10^6^ cells were used for CD marker staining and flow cytometry analysis.

#### CD marker staining for hHSCs

To determine the differentiation status of hHSCs after 11 days, lineage-specific, CD marker staining was performed as described elsewhere [37]. Differentiation into the erythroid lineage was shown with CD71 and CD235a, granulopoesis with CD11b and CD15 and megakaryopoesis with CD41a and CD42. Antibodies and isotype controls were obtained from BD and Beckmann Coulter, Germany. Staining was performed in 50 µL PBS^−^ for 15 min at 4 °C. The cells were washed with PBS^−^ and analyzed with flow cytometry.

#### Induction of differentiation for hMSCs

hMSCs were seeded in 6-well tissue culture plates (cytochemical staining) or 12-well tissue culture plates (RNA isolation, both Greiner Bio one, Frickenhausen, Germany) at 2 × 10^4^ cells/cm^2^ for adipogenic or 0.5 × 10^4^ cells/vm^2^ for chondrogenic and osteogenic differentiation. Cells for adipogenic differentiation were cultivated in α–MEM until confluence and then differentiation was induced with Adipogenic Induction Medium (Lonza, Cologne, Germany). The medium was changed twice a week, altering between adipogenic induction medium and adipogenic maintenance medium (Lonza, Cologne, Germany) for 3 weeks, followed by one week of cultivation in adipogenic maintenance medium. Osteogenic and chondrogenic differentiation was induced using NH Osteo Diff Medium/NH Chrondro Diff Medium (Miltenyi Biotec Bergisch Gladbach, Germany); the medium was changed twice a week. Analysis was performed after 24 days of cell culture.

Before starting the differentiation, all cells were incubated with 300 µg/mL nanoparticles for 24 h and non-treated samples served as a control. All experiments were also performed in α–MEM without differentiation factors (non-differentiated samples).

#### Cytochemical staining of hMSCs

Osteogenic differentiation was verified by detection of alkaline phosphatase activity using SIGMAFAST^TM^ BCIP^®^/NBT tablets. Briefly, incubation with a solution containing an alkaline phosphatase substrate results in a dark purple dye deposit, indicating the sites of alkaline phosphatase activity in cells differentiated into the osteogenic lineage. The extracellular matrix developed during chondrogenic differentiation was detected by staining with Loeffler´s methylene blue (Carl Roth, Karlsruhe, Germany). Adipogenic differentiation was detected by the lipophilic dye Oil Red O (Sigma Aldrich), which stained the lipid droplets of adipocytes deep red. Hematoxylin–Harris was used for counter staining which stained the cell nuclei blue.

#### Quantitative real time PCR (qPCR)

Total RNA was extracted from hHSCs or hMSCs using the RNeasy Kit (Qiagen, Hilden, Germany) according to the manufactures instructions. For better cell dissociation, Qiashredder columns (Qiagen, Germany) were used. RNA was transcribed using iScript cDNA Synthesis Kit (Bio-Rad, Germany). The concentration and quality of RNA and cDNA was determined with a NanoDrop 8000 spectrometer (Nanodrop, Wilmington, USA). qPCR reactions were done with an iQ SYBR^®^ green Supermix system (Bio-Rad) using the CFX96 Real-Time PCR Detection System (Bio-Rad) with the following program: initial denaturation at 95 °C for 3 min, followed by 40 cycles of PCR (95 °C for 10 s, annealing temperature (see Supporting Information File 1, Tables S2 and S3) for 10 s and 72 °C for 30 s and ending with a melting curve analysis (65 °C to 95 °C, increment 0.5 °C for 5 min). The primer sequences are given in Supporting Information File 1, Tables S2 and S3. The primers were used at a final concentration of 25 pmol/20 µL reaction. The primer mix for 18s RNA was purchased from Tataa Biocenter (Gothenburg, Sweden) and used in the recommended concentration. Expression of 18s RNA was used to normalize the gene expression level in hHSCs. For hMSCs, expression of glyceraldehyde-3-phosphate dehydrogenase (GAPDH) and β2 microglobulin (B2M) was used to normalize the gene expression level. The relative difference in the expression level was calculated using the ΔΔC_T_ method, setting the expression in the d0 sample as 1. The relative expression was used if d0 samples did not show expression of the used marker gene and was calculated with the ΔC_T_ method, setting the expression in the sample without particles to 1.

#### HTRF Assay

The levels of IL-8 in the cell culture supernatant were quantitatively investigated with Human IL-8 HTRF^®^ Assays (Cisbio Bioassays, Codolet, France) using the cell-supernatant protocol as recommended by the manufacturer. In brief, 0.5 × 10^6^ cells were seeded in 12-well plates and incubated with 300 µg/mL nanoparticles for 24 h in basal medium. Afterwards, the cells were washed 3 times with PBS^−^ supplied with fresh medium (basal medium supplemented with 50 ng/mL SCF and Flt) and cultured for 5 days. The supernatants were collected and stored at −80 °C until analysis. HTRF^®^ was performed in 384-well, low volume, flat bottom plates (Corning) according to the manufactures instructions in a total volume of 20 µL. The fluorescence signal was measured with an Infinite^®^ M 1000 (Tecan, Germany). The acquisition parameters were chosen according to the manufacturer’s manual (donor settings: top fluorescence mode, excitation 317 ± 0 nm, emission 620 ± 50 nm, integration time 500 µs, lag time 60 µs; acceptor settings the same except emission 665 ± 50 nm). Data analysis was performed with Magellan V6.6 software (Tecan) using a calibration curve which was included in the kit.

#### Statistical analysis

The qPCR and HTRF data were analyzed with one-way analysis of variance (ANOVA) followed by Tukey’s Multiple Comparison Test (GraphPad Prism 5, GraphPad Software Inc., USA). Statistical differences of p < 0.05 were considered significant. Significance is indicated in the figures as follows: p > 0.05 ns (not significant), p < 0.05 *, p < 0.01 **, and p < 0.001 ***.

## Supporting Information

File 1 Additional experimental results and material characterization.
The file contains several additional results complementing the results in the main text.
