# Supplementary material for: Hematopoietic and mesenchymal stem cells: polymeric nanoparticle uptake and lineage differentiation
Source: Beilstein J Nanotechnol. 2015 Feb 5;6:383–95. doi: 10.3762/bjnano.6.38 (PMC4362397; doi:10.3762/bjnano.6.38)
Supplement: File 1 — Additional experimental results and material characterization. The file contains several additional results complementing the results in the main text. [file Beilstein_J_Nanotechnol-06-383-s001.pdf]

Supporting Information

for

Hematopoietic and mesenchymal stem cells: polymeric nanoparticle uptake and lineage differentiation

Ivonne Brüstle[1], Thomas Simmet[2], Gerd Ulrich Nienhaus[3,4,5], Katharina Landfester[1], Volker Mailänder*[1,6]

Address: [1]Max-Planck-Institute for Polymer Research, Ackermannweg 10, 55128 Mainz, Germany, [2]Institute of Pharmacology of Natural Products & Clinical Pharmacology, Ulm University, Helmholtzstraße 20, 89081 Ulm, Germany, [3]Institute of Applied Physics, Karlsruhe Institute of Technology (KIT), Wolfgang-Gaede-Straße 1, 76131 Karlsruhe, Germany, [4]Institute of Toxicology and Genetics (ITG), Karlsruhe Institute of Technology (KIT), Hermann-von-Helmholtz-Platz 1, 76344 Eggenstein-Leopoldshafen, Germany, [5]Department of Physics, University of Illinois at Urbana-Champaign, Urbana, Illinois 61801, USA and [6]3rd Department of Medicine (Hematology, Oncology, and Pneumology), University Medical Center of the Johannes Gutenberg-University Mainz, Langenbeckstraße 1, 55131 Mainz, Germany,

Email: Volker Mailänder* - volker.mailaender@mpip-mainz.mpg.de

*Corresponding Author

Additional experimental results and material characterization

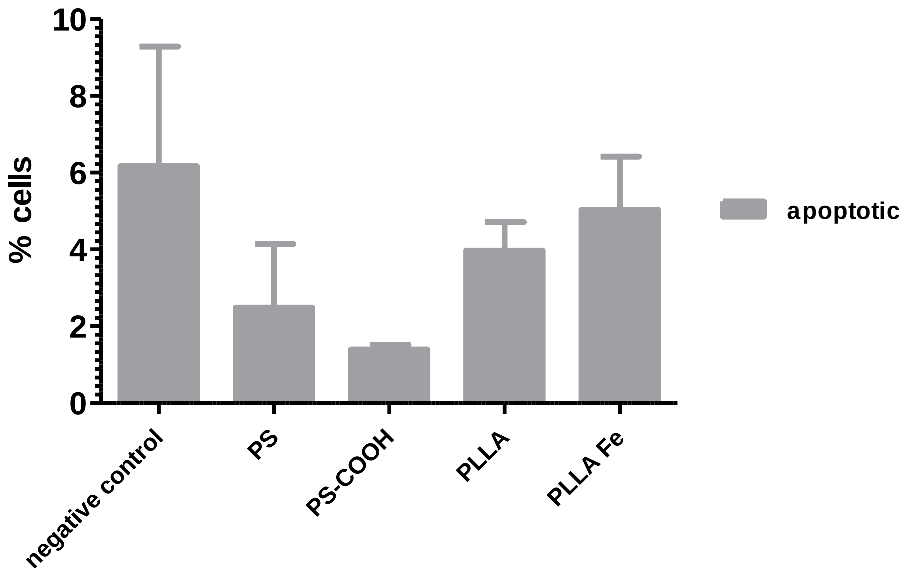

Figure S1: Amount of apoptotic cells after incubation with indicated nanoparticles. As this is low and the apoptotic fraction is not enhanced in quantity the investigated nanoparticles can be considered to be safe.

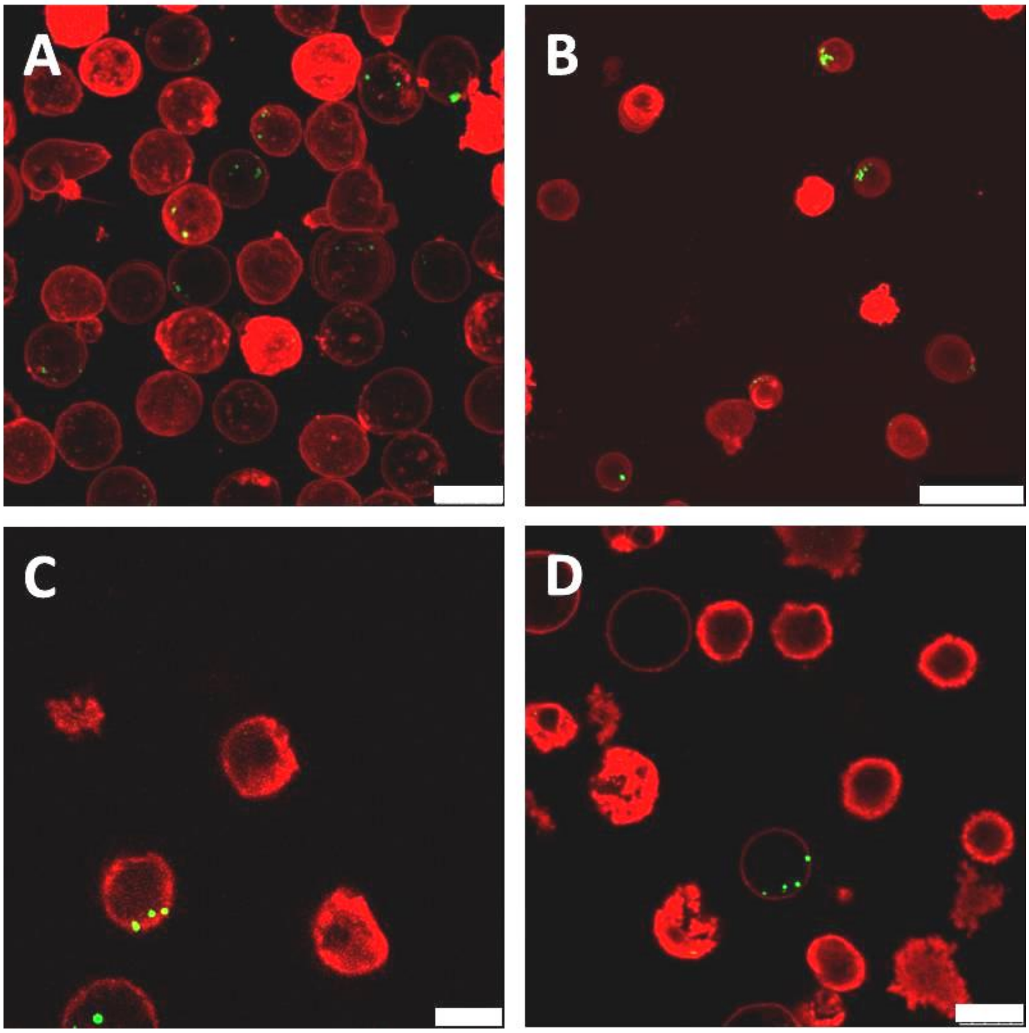

Figure S2: (A) PS (B) PS–COOH (C) PLLA (D) PLLA–Fe particles in hHSCs. The cell membrane is pseudo-colored in red, nanoparticles are pseudo-colored in green. Scale bars are 10 µm for (A), (C) and (D); for (B) scale bar is 25 µm.

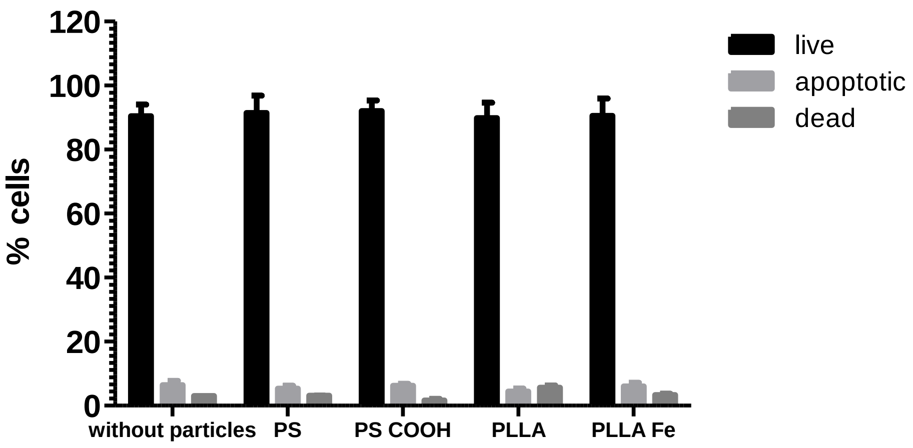

Figure S3: Cytotoxicity of the particles after 24 h incubation with 300 µg/mL nanoparticles analyzed by 7-AAD staining and flow cytometry.

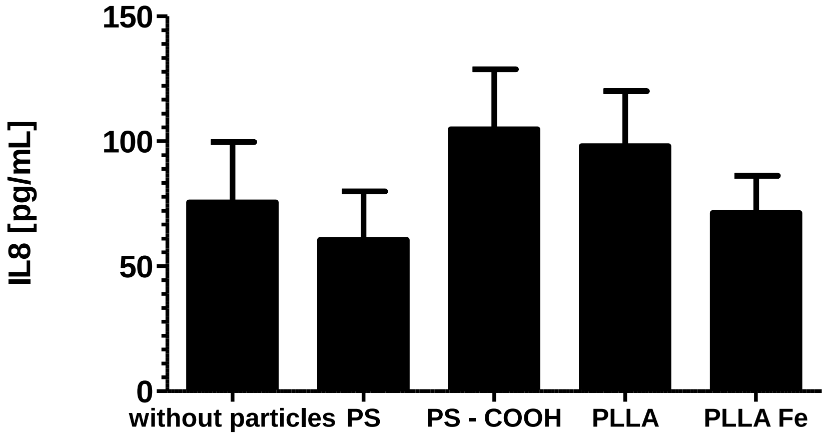

Figure S4: IL-8 secretion of hHSCs. Measurement was done in the supernatant of cells that have been incubated for 24 h with 300µg/mL nanoparticles, washed three times with PBS$^-$ and cultivated for 5 days in medium with SCF and Flt at the end of this period. The differences were not statistically significant.

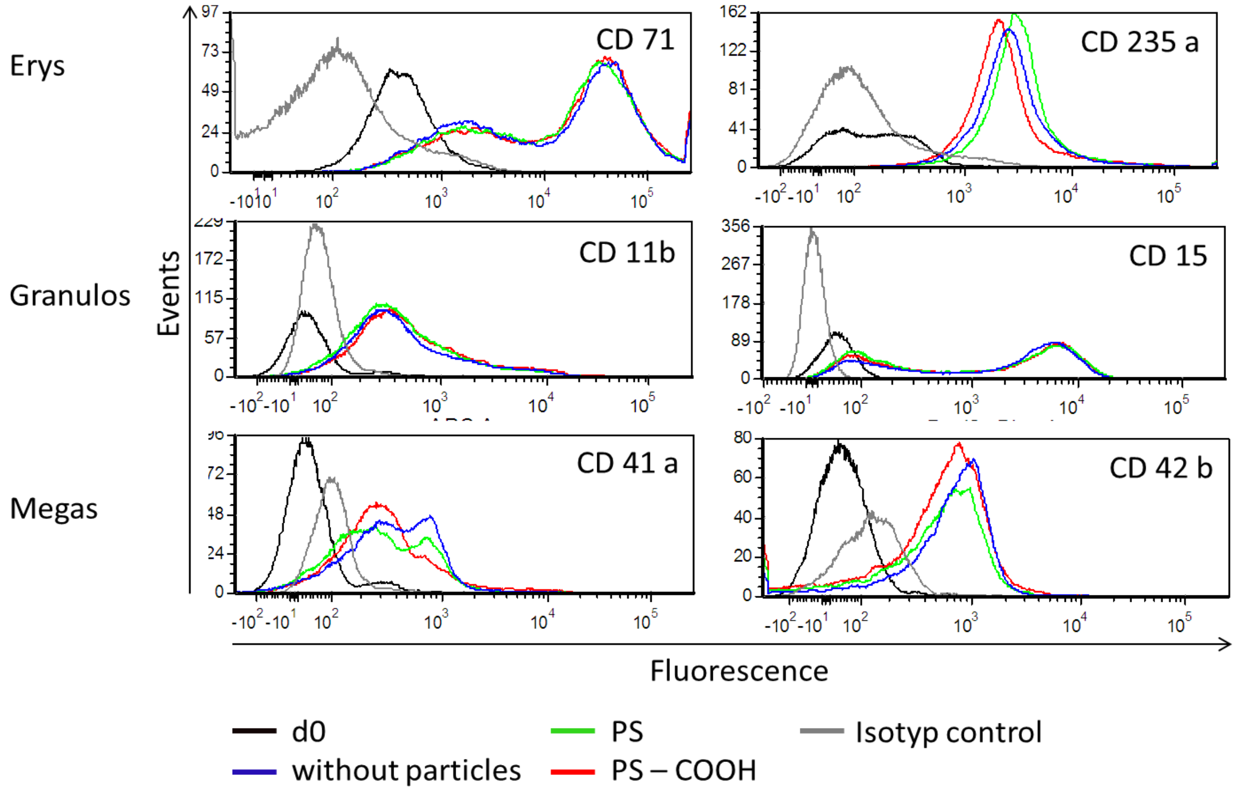

Figure S5: CD marker staining polystyrene particles. For erythropoiesis CD71 and CD235a were determined, while for granulopoesis CD11b and CD15 were chosen. Megakaryopoesis was demonstrated by CD41a and CD42b.

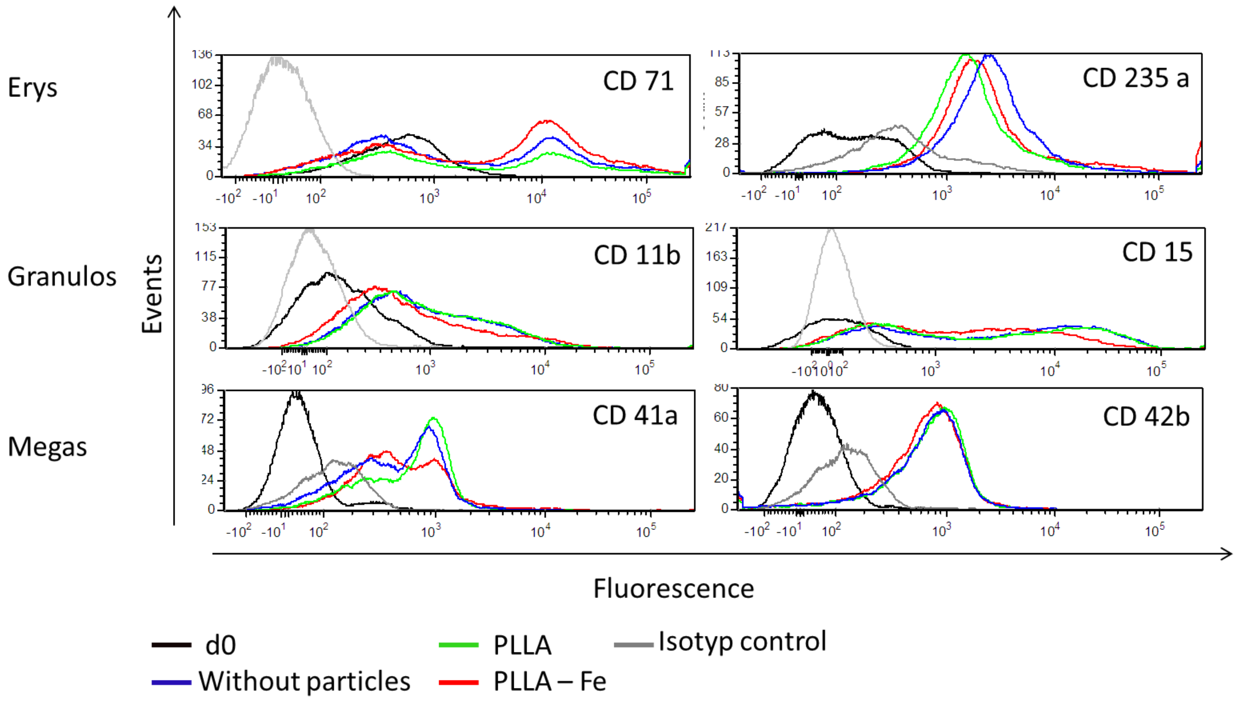

Figure S6: CD marker staining polylactide particle. For erythropoiesis CD71 and CD235a were determined, while for granulopoesis CD11b and CD15 were chosen. Megakaryopoesis was demonstrated by CD41a and CD42b.

Table S1: Supplements used for hHSC lineage differentiation. SCF: stem cell factor; Flt3: Flt-3/Flk-2 ligand; EPO Erythropoietin, TPO Thrombopoietin.

	Erythropoesis	Granulopoesis	Megakaryopoesis
supplements	SCF (50 ng/mL)<br>Flt3 (50 ng/mL)<br>Il3 (10 ng/mL)<br>Epo (10 U/mL)	SCF (50 ng/mL)<br>Flt3 (50 ng/mL)<br>Il3 (10 ng/mL)<br>G-CSF (10 ng/mL)<br>GM-SCF (10 ng/mL)	SCF (50 ng/mL)<br>Flt3 (50 ng/mL)<br>TPO (20 ng/mL)

Table S2: Primer sequences used for qPCR analysis of hHSCs.

Target	Forward	Reverse	Product size (bp)	Annealing Temp °C
RAP1GA1 (RAP1, GTPase activating protein 1)	ACAGGTCTAGTGCCTG AGGG	CCCTTGGGGTGGACA AG	90	63
GPE (Glycophorin E)	CACACCAGTGGTACTT GATGC	TGCACTAACTTCAGG AGCCA	110	60
CEACAM (Carcinoembryonic antigen related cell adhesion molecule1)	GAGAGGCCATTTTCTT GTGG	GGGACGTATTGGTGT GAGGT	104	63
IL2RA (Interleukin 2 receptor alpha)	TAGGCCATGGCTTTGA ATGT	ACTGCTCACGTTCATC ATGG	96	63
TREM2 (Triggering receptor expressed on myeloid cells)	AGTCATAGGGGCAAGA CACC	CCGGCTGCTCATCTT ACTCT	107	60

Table S3: Primer sequences used for qPCR analysis of hMSCs.

Target	Forward	Reverse	Product size (bp)	Annealing Temp °C
GAPDH (Glyceraldehyde 3-phosphate dehydrogenase)	AATGAAGGGGTCATT GATGG	AAGGTGAAGGTCGGA GTCAA	108	60
B2M (β2 microglobulin)	TCTCTGCTGGATGAC GTGAG	TAGCTGTGCTCGCGC TACT	90	60
FABP4 (fatty acid binding protein 4)	TGATGATCATGTTAGG TTTGGC	TGGAAACTTGTCTCCA GTGAA	106	60
CIDE 3 (cell death-inducing DFFA-like effector c)	CAGTTGTGCCATCTTC CTCC	AAGGGCATCATGGCT TACAG	109	60
TIMP4 (TIMP metallopeptidase inhibitor 4)	GGCTCGATGTAGTTG CACAG	ACGCCTTTTGACTCTT CCCT	125	60
Osteopontin [1]	CTC AGG CCA GTT GCA GCC	GCC ACA GCA TCT GGG TAT TT	177	60
Alkaline phosphatase) 1	CCT CGG AAG ACA CTC TGA CC	CCA CCA AAT GTG AAG ACG TG	61	60

References

[1] Tautzenberger, A.; Lorenz, S.; Kreja, L.; Zeller, A.; Musyanovych, A; Schrezenmeier, H; Landfester, K; Mailänder, V.; Ignatius, A. *Biomaterials* **2010,** *31*,2064–2071.
